# In Silico Molecular Docking Studies and Xanthine Oxidase Inhibitory Activity of *Abies kawakamii* Leaf Extract and Its Constituent

**DOI:** 10.3390/ph19071100

**Published:** 2026-07-17

**Authors:** Chi-Ya Huang, Pei-Ling Yen, Li-Sheng Hsu, Jinn-Guan Low, Yu-Mei Huang, Shou-Ling Huang, Chun-Han Ko, Hui-Ting Chang

**Affiliations:** 1 Agricultural Technology Research Institute, Hsinchu 300110, Taiwan; r99625047@ntu.edu.tw; 2 Program in Specialty Crops and Metabolomics, Academy of Circular Economy, National Chung Hsing University, Nantou 54071, Taiwan; plyen@dragon.nchu.edu.tw; 3 School of Forestry and Resource Conservation, National Taiwan University, Taipei 10617, Taiwan; r97625041@ntu.edu.tw (L.-S.H.); r13625045@ntu.edu.tw (J.-G.L.); chunhank@ntu.edu.tw (C.-H.K.); 4 Institute of Environmental and Occupational Health Sciences, National Taiwan University, Taipei 10055, Taiwan; d95841007@ntu.edu.tw; 5 Instrumentation Center, College of Science, National Taiwan University, Taipei 10617, Taiwan; shouling@g.ntu.edu.tw

**Keywords:** *Abies kawakamii*, essential oil, hyperuricemia, terpenoids, xanthine oxidase inhibitory activity

## Abstract

Gout is a metabolic disorder associated with abnormal purine metabolism and persistent hyperuricaemia, which promotes formation and deposition of monosodium urate crystal in joints, leading to acute arthritis and impaired quality of life. This study aimed to evaluate the xanthine oxidase inhibitory activity of ethanolic leaf extract of *Abies kawakamii* and its fractions. The protocol of bioassay-guided fractionation was employed to isolate active compounds from leaf extract. Maltol was identified as the major active compound from the ethyl acetate fraction (EAF). Maltol inhibited xanthine oxidase with IC_50_ values of 33.18 and 26.67 μg/mL against xanthine and hypoxanthine, respectively, significantly lower than the crude extract (152.04 and 136.57 μg/mL) and EAF (66.70 and 48.76 μg/mL). Enzyme kinetic analyses further showed that EAF inhibited xanthine oxidase through a competitive inhibition mechanism toward both substrates. Molecular docking analysis suggested that maltol may interact with the active-site region of xanthine oxidase, showing a binding affinity of −6.5 kcal/mol, slightly weaker than that of allopurinol, with no predicted hepatotoxicity based on in silico analysis. Our findings indicate that *A. kawakamii* leaf extract and its constituent, maltol, could be further explored as a natural approach for gout management.

## 1. Introduction

Gout is a disorder associated with abnormal purine metabolism caused by elevated uric acid levels (hyperuricemia), which promote monosodium urate crystals formation and deposition in joints [1]. The typical symptoms of acute gout are characterized by intense joint pain, accompanied by swelling and redness, thereby affecting patients’ quality of life. During purine catabolism, purines derived from dietary intake and endogenous metabolism are sequentially converted into hypoxanthine and xanthine through a series of enzymatic processes. Xanthine oxidase subsequently catalyzes the oxidation of hypoxanthine to xanthine and finally converts xanthine into uric acid [1,2,3,4,5]. Insulin resistance would increase the body weight and serum uric acid level. Gout can be classified into four clinical features, including asymptomatic hyperuricemia, acute gouty arthritis, chronic tophaceous gout, and intercritical gout. Modifications of the dietary and lifestyle of gout patients to reach the ideal weight, and both insulin resistance and serum uric acid levels could decrease [4,5,6,7]. In addition, allopurinol has been widely used as a xanthine oxidase inhibitor for controlling gout and hyperuricemia, while more recently developed medications include febuxostat, topiroxostat, and uricosuric agents such as probenecid. Certain anti-gout medications may be associated with adverse reactions; for example, febuxostat has been reported to induce diarrhea, elevation of liver enzymes, and nausea [8,9,10].

Many researchers have proven that plant natural products exhibited a lot of bioactivities, including antioxidant, antimelanogenic, antimicrobial, xanthine oxidase inhibitory, tyrosinase inhibitory, anti-tumor, anti-inflammatory, larvicidal, and antidepressant activities [11,12,13,14,15,16,17,18,19]. Researchers have increasingly investigated plant-derived natural products as potential sources of xanthine oxidase inhibitors [20,21,22,23,24,25]. The twig extract of *Cinnamomum cassia* demonstrated inhibitory activity against xanthine oxidase [26]. Extracts obtained from *Tetracera scandens*, *Blumea balsamifera*, *Caesalpinia sappan*, and *Chrysanthemum sinense* demonstrated inhibitory activity toward xanthine oxidase [27]. Phytic acid, which is widely distributed in cereals and nuts, can suppress uric acid formation using xanthine as the substrate, with an IC_50_ of nearly 30 mM [28].

The genus *Abies* (Pinaceae), Fir, includes 50–60 species is widely distributed in South Europe, North America and East Asia. Natural products from Fir have been used traditionally to support respiratory health, pulmonary diseases, colds, and rheumatic diseases antitumor activity against adenocarcinoma [29,30,31,32,33]. *Abies kawakamii* is known as Taiwanese Fir or Formosan Fir, is an evergreen coniferous tree and the endemic species in Taiwan. The genus *Abies* has a long history of ethnomedicinal use in traditional medicine. Its reported ethnomedicinal bioactivities include anti-inflammatory, analgesic, antitussive, antimicrobial, antioxidant, antidiabetic, etc., and have long histories of use in traditional medicine [30,31,33,34,35,36]. Its decoctions and extracts were traditionally used to treat rheumatism, joint inflammation, and symptoms associated with gout [30,31]. However, there is still very limited research on the bioactive effects of extracts from *A. kawakamii*, and no paper reported the xanthine oxidase inhibition potential of *A. kawakamii* leaf extract and its fractions.

This study investigated the xanthine oxidase inhibition potential of *A. kawakamii* leaf extract and its fractions. The phytochemical constituents present in leaf extract, active fractions, and active compound were isolated and identified by liquid chromatography and spectroscopies. An enzyme kinetic study and in silico molecular docking analyses were applied to elucidate the mechanism of active specimens against xanthine oxidase.

## 2. Results and Discussion

### 2.1. Xanthine Oxidase Inhibition Effects of A. kawakamii Leaf Extract and Its Fractions

*A. kawakamii* leaf extract was prepared by ambient solvent extraction and subsequently separated into four fractions (HXF, EAF, BUF, and HOF) through liquid–liquid partitioning. Table 1 summarizes the xanthine oxidase inhibitory activities of *A. kawakamii* leaf extract, its fractions, and allopurinol as the positive control at 100 μg/mL against hypoxanthine and xanthine substrates. Using hypoxanthine and xanthine as substrates, the leaf extract exhibited inhibition rates of 44.40% and 31.07%, respectively. Complete inhibition (100%) against both substrates was observed for allopurinol and the ethyl acetate fraction. The EAF showed inhibitory activities of 100% and 80.82% toward hypoxanthine and xanthine, respectively. BUF showed the lowest activity, whereas HXF displayed no detectable inhibition.

**Figure 1:**
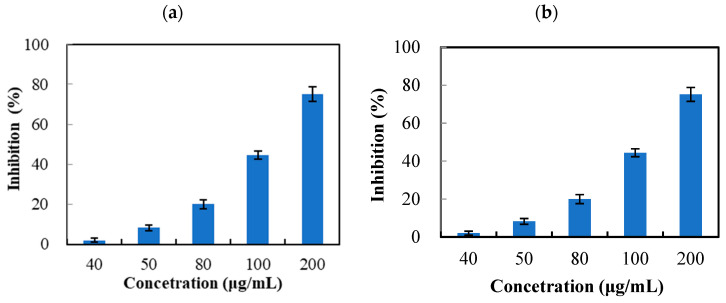
Xanthine oxidase inhibitory effects of *A. kawakamii* extract using (**a**) hypoxanthine and (**b**) xanthine as substrates. Data are presented as mean ± SD (*n* = 3).

*A. kawakamii* leaf extract suppressed xanthine oxidase activity in a dose-dependent manner when hypoxanthine and xanthine were used as substrates (Figure 1), with the IC_50_ values of 136.57 and 152.04 μg/mL, respectively (Table 2). EAF showed the strongest inhibitory activity against xanthine oxidase, with the IC_50_ values of 48.76 μg/mL for hypoxanthine and 66.70 μg/mL for xanthine (Table 2). Results revealed that *A. kawakamii* leaf extract and EAF possess considerable xanthine oxidase inhibitory potential.

Abdulhafiz et al. reported *Alocasia longiloba* (Keladi Candik), traditional medicine in Malaysia, extracts were found to exhibit the high xanthine oxidase inhibitory activity, with IC_50_ values range of 42.71 to 51.32 µg/mL [25]. Xanthine oxidase inhibitory activities were reported in both aqueous and ethanolic extract of *Pistacia integerrima* leaves, with IC_50_ values of 85 and 60 μg/mL, respectively, when hypoxanthine served as the substrate. [37]. Xanthine oxidase inhibitory activities were observed in the bark and leaf extracts of *Erythrina variegata*, yielding IC_50_ values of 52.75 and 84.75 μg/mL, respectively, when xanthine served as the substrate. [38]. The present results demonstrated that *A. kawakamii* leaf extract and its EAF possessed the xanthine oxidase inhibitory activity. In addition, EAF showed significantly higher activity than the other tested specimens (*p* < 0.05).

### 2.2. Structural Characterization of Maltol

**Figure 2:**
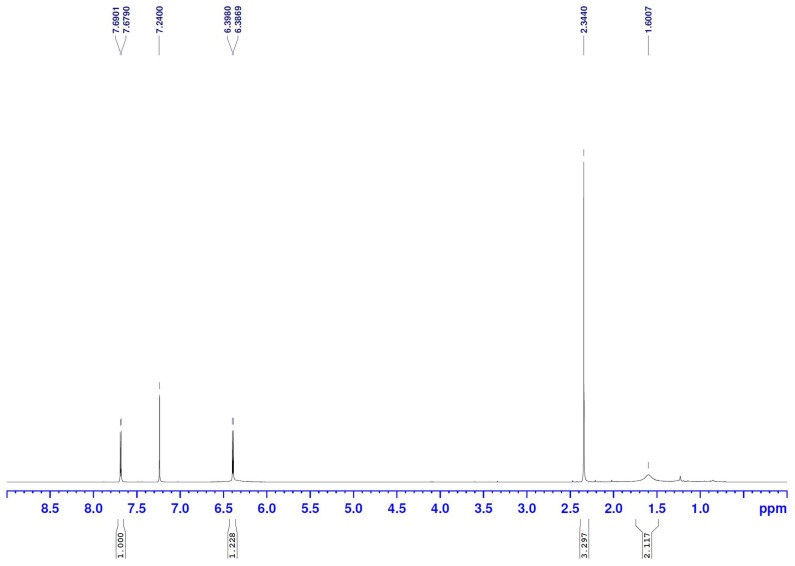
^1^H-NMR spectral data of maltol (CDCl_3_, 500 MHz).

**Figure 3:**
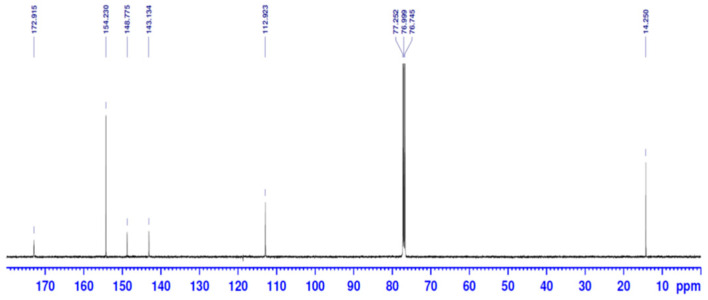
^13^C-NMR spectral data of maltol (CDCl_3_, 125 MHz).

**Figure 4:**
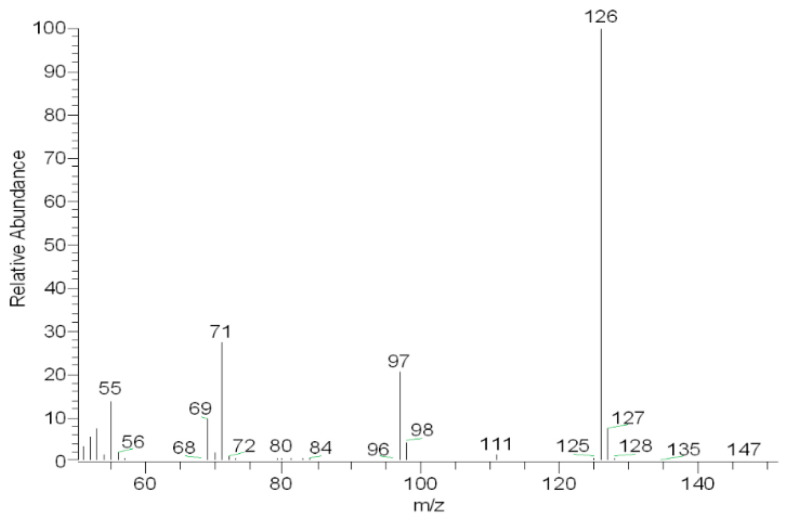
Mass spectrum of compound M.

**Figure 5:**
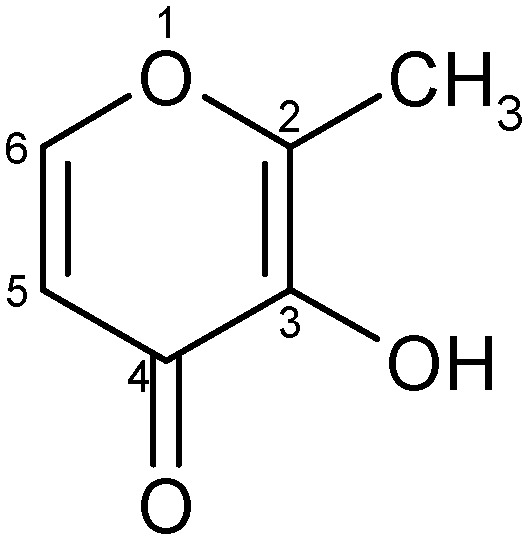
Chemical structure of maltol.

Compound M from EAF was characterized as maltol using NMR (Figure 2 and Figure 3) and MS spectroscopy (Figure 4). Table 3 presents the ^1^H and ^13^C NMR data of maltol. White crystal needle; mp: 161–163 °C; UV (MeOH) λmax (log ε): 275, and 322 nm; ^1^H NMR (CDCl_3_, 500 MHz) δ 7.69 (1H, d, J = 5.55 Hz), 6.39 (1H, d, J = 5.55 Hz), 2.34 (3H, s, C2-CH_3_). ^13^C NMR (CDCl_3_, 125 MHz) δc 172.9 (C-4), 154.2 (C-6), 148.8 (C-2), 143.1 (C-3), 112.9 (C-5). NMR spectra were in agreement with the literature [39,40]. Figure 4 was the mass spectrum of compound M. EI-MS *m*/*z*: 55 (14), 69 (9), 71 (27), 97 (20), 126 (M+, 100), molecular formula C_6_H_6_O_3_. Figure 5 shows the chemical structure of maltol (3-hydroxy-2methyl-4H-pyran-4-one).

Maltol was also isolated from the same genus, *Abies sibirica* (Siberian Fir), *Abies pindrow*, *Abies fraseri*, and *Abies nebrodensis* leaf extracts [40,41,42,43]. It is also found in other species and baked products. Maltol is a naturally occurring compound, with sweet, and caramel-like scents, resulting from the heterocyclic structure. The bioactivities of maltol include antimicrobial, antioxidant, ROS scavenging, anti-inflammatory, anticancer, etc. [42,44,45,46]. Maltol is used as a safe and reliable flavor enhancer, and is designated in the U.S. and Europe with the E number E636 and the CAS number 118-71-8 [42,44,45,46,47].

### 2.3. Xanthine Oxidase Inhibitory Activity and Enzyme Kinetic Study of Ethyl Acetate Fraction and Its Constituents

**Figure 6:**
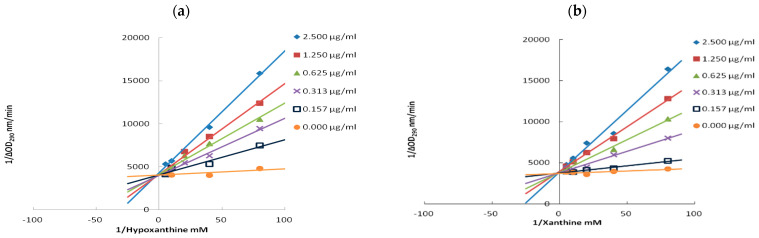
Kinetic inhibition profiles of allopurinol toward xanthine oxidase using (**a**) hypoxanthine and (**b**) xanthine as substrates.

The major types of enzyme inhibition include noncompetitive, uncompetitive, competitive, and mixed types. To validate the experimental system, an enzyme kinetic study was first conducted using allopurinol as a positive control (Figure 6). With both hypoxanthine and xanthine as substrates, the Lineweaver–Burk plots of allopurinol displayed identical y-intercepts but progressively steeper slopes as the inhibitor concentration increased. Table 4 summarizes the kinetic parameters of allopurinol against xanthine oxidase, showing an increased *K_m_* while *V_max_* remained unchanged. Accordingly, competitive inhibition of xanthine oxidase by allopurinol contributed to the suppression of uric acid formation. Competitive-type inhibition by allopurinol has previously been reported by Huang et al. and Chen et al., which is consistent with our data [48,49]. It manifested that allopurinol showed a strong preference for interacting with free xanthine oxidase, consequently hindering substrate interaction with the enzyme.

**Figure 7:**
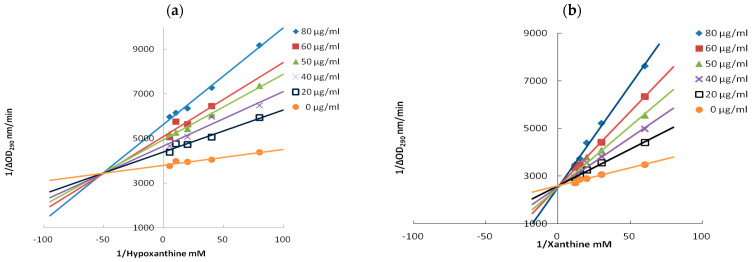
Kinetic inhibition profiles of ethyl acetate fraction toward xanthine oxidase using (**a**) hypoxanthine and (**b**) xanthine as substrates.

Figure 7 shows the Lineweaver–Burk plots of EAF with two substrates. Kinetic parameters of EAF were listed in Table 5. In the presence of EAF, *K_m_* increased while *V_max_* decreased when hypoxanthine was used as the substrate, indicating a mixed-type inhibition pattern. In contrast, the use of xanthine as the substrate resulted in an increased *K_m_* and without a change in *V_max_*, suggesting competitive inhibition.

**Figure 8:**
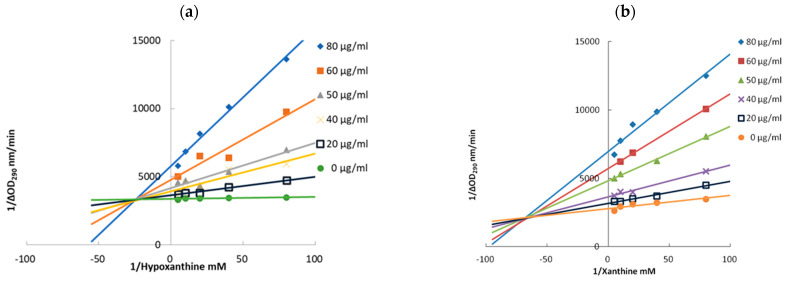
Kinetic inhibition profiles of maltol toward xanthine oxidase using (**a**) hypoxanthine and (**b**) xanthine as substrates.

The inhibitory mode of maltol toward xanthine oxidase was further clarified by kinetic analysis, with the corresponding Lineweaver–Burk plots and kinetic constants presented in Figure 8 and Table 6. Maltol treatment altered both kinetic parameters, as reflected by an increased *K_m_* and a decreased *V_max_*. It demonstrated that maltol suppresses uric acid production through a mixed-type inhibition mechanism. Similar inhibitory behavior has been reported for quercetin, a multifunctional flavonoid, which inhibits xanthine oxidase through both competitive and noncompetitive components [48,49,50].

To further contextualize these findings, the inhibitory activity of the present samples was compared with previously reported natural products. Xanthine oxidase inhibitory activity was detected in *Pistacia integerrima* leaf extract and its fractions, where quercetin-3-*O*-D-glucopyranoside, rutin, and apigenin showed IC_50_ values ranging from 5.75 to 61 μg/mL for hypoxanthine as substrate [37]. Xanthine oxidase inhibitory activity was identified in the ethyl acetate extract of snake fruit (*Salacca edulis*), in which 2-metyl ester-1*H*-pyrrole-4-carboxilyc acid exhibited the strongest activity with an IC_50_ value of 48.86 μg/mL for xanthine as substrate [51]. In the present study, maltol showed IC_50_ values of 26.67 μg/mL and 33.18 μg/mL using hypoxanthine and xanthine as substrates, respectively, indicating a moderate inhibitory effect comparable to other natural products.

### 2.4. Molecular Docking of Maltol with Xanthine Oxidase

**Figure 9:**
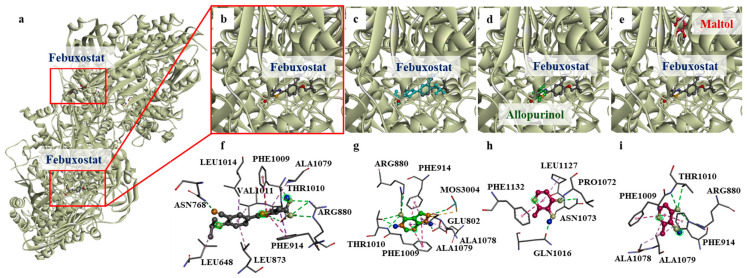
Molecular docking of maltol within the active site of xanthine oxidase. (**a**) Representation of xanthine oxidase (PDB ID: 1N5X), showing febuxostat located in both chain A and chain B of xanthine oxidase co-crystallized structure. (**b**) Enlarged view of the active site in chain A with the co-crystallized febuxostat. (**c**–**e**) Overlay of docked poses and the co-crystallized febuxostat within the active site, showing (**c**) febuxostat (cyan), (**d**) allopurinol (green), and (**e**) maltol (red). (**f**–**i**) Interaction of (**f**) febuxostat, (**g**) allopurinol, and (**h**,**i**) maltol with residues in the xanthine oxidase active site. Green dashed lines indicate hydrogen bonds; pink lines indicate π–π and π–alkyl interactions; purple lines indicate π–sigma interactions; gold lines indicate π–sulfur interactions; and red lines represent unfavorable donor–donor interactions.

The mixed-type inhibition pattern observed for maltol suggests that its interaction with xanthine oxidase may involve more complex binding behavior than simple competitive inhibition [52]. However, enzyme kinetic analysis alone cannot definitively determine the underlying molecular interaction mechanism. Molecular docking analysis further indicated possible interactions between maltol and residues located within the active-site channel. The xanthine oxidase structure (PDB ID: 1N5X, Figure 9a,b) was used to predict the binding site of maltol. To validate the docking protocol, febuxostat was first removed from the co-crystallized structure and subsequently redocked into the active site. The redocked pose closely overlapped with the co-crystallized conformation, with only minor differences in the orientation of functional groups (Figure 9c). Among the 20 independent redocking runs performed for febuxostat, the pose with the lowest root-mean-square deviation (RMSD) relative to the crystallographic febuxostat pose was selected as the best redocked pose. This pose yielded an RMSD of 0.911 Å, supporting the validity of the docking protocol. Across the 20 independent redocking runs, febuxostat showed a mean binding affinity of −8.5 ± 0.3 kcal/mol. In the crystallographic binding pose, febuxostat interacted with ASN768, ARG880, and THR1010 through hydrogen bonding, along with π–alkyl interactions with LEU873 and VAL1011, and a π–sigma interaction with LEU1014 and PHE914 (Figure 9f and Figure S1a).

Allopurinol was readily accommodated within the same active site (Figure 9d), although its binding affinity (−7.0 ± 0.1 kcal/mol) was weaker than that of febuxostat (−8.5 ± 0.3 kcal/mol). It interacted with GLU802, ARG880, and THR1010 through hydrogen bonding and exhibited π–π interactions with PHE914 and PHE1009 (Figure 9g and Figure S1b).

For maltol, the predominant docking pose (11 of 20 runs) was in a peripheral region, involving hydrogen bonding with LEU1127, ASN1073, and GLN1016, along with π–π and π–alkyl interactions with PHE1132 and PRO1072, respectively (Figure 9h and Figure S1c). This binding mode was positioned farther from the co-crystallized febuxostat (Figure 9e), and an unfavorable acceptor–acceptor interaction was observed between the hydroxyl group of maltol and ASN1073. In the remaining poses, alternative hydrogen bonding with THR1010 and ARG880 was identified (Figure 9i and Figure S1d). Notably, these residues are key components of the canonical active site, suggesting that maltol may adopt a secondary conformation within the Mo-pterin active-site channel. Despite its shifted binding position, maltol still exhibited a stable interaction with a binding affinity of −6.5 ± 0.1 kcal/mol.

The Mo-pterin active channel of xanthine oxidase is a key substrate-access tunnel leading to the deep catalytic center and is lined by residues such as ARG880, PHE914, PHE1009, and THR1010 [53]. Our docking results suggest that maltol is capable of accessing this active-site channel, as approximately 50% of the docked poses were located within the Mo-pterin channel. However, compared with allopurinol, maltol showed weaker binding affinity and less consistent localization within this region. In contrast, allopurinol occupied the active-site channel in 19 of 20 runs and formed more favorable interactions with the residues in this key substrate-access tunnel. Febuxostat was also able to bind within the same site and displayed more extensive interactions with xanthine oxidase; however, it occupied this position in only 8 of 20 runs, likely reflecting conformational variability in docking rather than reduced binding capability. These observations are consistent with the higher IC_50_ and lower inhibitory potency of maltol. However, the docking results alone are insufficient to establish the inhibition mechanism.

Compared with flavonoids, stilbenes, or other synthetic xanthine oxidase inhibitors [54,55], maltol is structurally smaller and less capable of forming multiple stabilizing interactions with active-site residues. Nevertheless, maltol was predicted to interact with critical residues such as ARG880 and THR1010. These interaction patterns may partially explain the enzyme kinetic results, in which allopurinol exhibited competitive inhibition whereas maltol displayed a mixed-type inhibition pattern. Overall, the docking results suggest that allopurinol and febuxostat are more likely to occupy the canonical active-site channel of xanthine oxidase, whereas maltol may act as a milder modulator with a more complex interaction pattern than simple competitive inhibition, possibly due to weaker and less stable interactions within the active-site channel. Although these docking findings may help explain the observed inhibition patterns, they should still be interpreted with caution because they represent predicted binding modes rather than experimentally validated mechanisms.

### 2.5. In Silico Predictions of Physicochemical, Pharmacokinetic, and Toxicological Properties of Maltol

To evaluate the potency of maltol as a potential xanthine oxidase inhibitor, its physicochemical properties, pharmacokinetics, and toxicity were predicted using the SwissADME and ProTox 3.0 platforms, respectively, with allopurinol used as a reference compound. The results showed that the Log S (ESOL) and Log P (o/w) values of maltol were −1.17 and 0.55, respectively (Table S2), indicating high aqueous solubility and suggesting favorable bioavailability. Although its properties were slightly different, they remained comparable to those of allopurinol (Log S (ESOL) = −0.93; Log P (o/w) = 0.01). Consistently, both compounds exhibited an identical predicted bioavailability score of 0.55 in SwissADME.

Evaluation based on the Lipinski’s Rule of Five, Ghose filter, and Veber rule revealed three violations for maltol, including a molecular weight below 160, a molar refractivity of 31.97 (below 40, Table S2), and fewer than 20 atoms. Similarly, allopurinol also exhibited three violations, including a molecular weight below 160, a molar refractivity of 34.51 (below 40, Table S2), and fewer than 20 atoms. Although such violations may generally limit drug-likeness, these deviations are primarily associated with the small molecular size of both compounds. Notably, allopurinol, an approved drug, exhibits similar violations, suggesting that these features are unlikely to substantially impair the therapeutic efficacy of maltol as an orally active agent.

Maltol and allopurinol did not exhibit inhibitory activity against the five major cytochrome P450 isoforms (Table S3), suggesting a low potential for drug–drug interactions. In addition, it showed high gastrointestinal absorption, indicating suitability for systemic therapeutic applications. Unlike allopurinol, maltol is predicted to cross the blood–brain barrier (BBB); therefore, its potential central nervous system (CNS) effects require further investigation. Maltol and allopurinol were also predicted not to be a P-glycoprotein (P-gp) substrate, suggesting a higher likelihood of absorption and retention.

Toxicity predictions indicated that maltol is inactive for hepatotoxicity, neurotoxicity, respiratory toxicity, cardiotoxicity, immunotoxicity, and cytotoxicity, while showing weak activity in nephrotoxicity, carcinogenicity, and mutagenicity endpoints (Table S4). Compared with allopurinol, which has been associated with hepatotoxicity, maltol exhibited a lower predicted risk of hepatotoxicity. As most compounds typically exhibit at least one predicted toxicological liability [54], maltol showed a relatively favorable in silico toxicity profile. However, these ADMET and toxicity predictions should be interpreted as preliminary screening results rather than definitive evidence of safety, as they are based on computational models and require further experimental validation. Taken together, these findings suggest that maltol may warrant further investigation for its potential as an alternative candidate.

## 3. Materials and Methods

### 3.1. Plant Material and Extraction

*Abies kawakamii*, 76 years old, was collected from the Duigaoyue Forest Working Unit, Experimental Forest of National Taiwan University. The collection source and laboratory records of the plant materials followed our previously published report [56]. Fresh leaves were subjected to two rounds of extraction with 95% ethanol at room temperature for 7 days each. After filtration, the solvent was removed under vacuum using a rotary evaporator [57]. The 95% ethanolic extraction yield of *A. kawakamii* leaves was 9.24% on a dry weight basis.

### 3.2. Liquid–Liquid Partition

The extract was further separated by liquid–liquid partitioning with solvents of progressively increasing polarity. Four fractions were subsequently obtained, including the *n*-hexane fraction (HXF), ethyl acetate fraction (EAF), *n*-butanol fraction (BUF), and aqueous fraction (HOF) [23,57,58]. The four fractions exhibited a decreasing trend in content levels: EAF (31.40%) > HOF (20.90%) > HXF (14.43%) > BUF (7.96%).

### 3.3. High Performance Liquid Chromatography

Bioactive fractions were further purified using semi-preparative high-performance liquid chromatography (HPLC; L-2130, Hitachi, Tokyo, Japan) equipped with a 9.4 × 250 mm Zorbax Sil column (5 μm). Separation was carried out under isocratic conditions with an *n*-hexane–ethyl acetate–acetone mixture (1:1:1, *v*/*v*) as the mobile phase at a flow rate of 1 mL/min, and chromatographic signals were monitored using a refractive index (RI) detector [58,59].

### 3.4. Isolation and Identification of Compounds

The isolated constituents were structurally characterized through spectroscopic analyses, including mass spectroscopy (MS; MAT-958, Finnigan, MA, USA), nuclear magnetic resonance spectroscopy (NMR; Bruker AVIII, Bruker Avance, Rheinstetten, Germany). The ^1^H-NMR and ^13^C-NMR spectra were acquired at 500 MHz and 125 MHz, respectively [59,60,61,62].

### 3.5. Xanthine Oxidase Assay

Xanthine oxidase inhibition was determined using in vitro spectrophotometric method [23,37,49]. Hypoxanthine and xanthine were separately employed as substrates for the assay. Briefly, xanthine oxidase solution (60 µL, 0.025 unit/mL; EC 1.1.3.22), 50 mM potassium phosphate buffer (117 µL, pH 7.8), and test sample (3 µL) were introduced into the 96-well plate for 10 min at 25 °C. Subsequently, 100 µL of substrate (0.15 mM xanthine or hypoxanthine) was added and incubated at 37 °C in the dark for 30 min. 20 µL of 1 N HCl was used to stop the enzymatic reaction. Absorbance at 290 nm was monitored using a microplate spectrometer (SPECTROstar Nano, BMG LABTECH, Offenburg, Germany). Allopurinol was included as a positive control, and each experiment was conducted in triplicate. Xanthine oxidase inhibition was calculated using the following equation:

Inhibition (%) = {[(A_control − A_control blank) − (A_sample − A_sample blank)]/[(A_control − A_control blank)]} × 100.

The IC_50_ was obtained from the dose–response curve and defined as the concentration required to inhibit 50% of xanthine oxidase activity.

### 3.6. Enzyme Kinetic Study

Lineweaver–Burk plots were applied to estimate the kinetic behavior of xanthine oxidase and to clarify how the tested samples affected the enzyme–substrate interaction. The enzyme concentration was fixed at 0.025 unit/mL, while the substrate concentration was adjusted from 0.0125 to 0.20 mM using either hypoxanthine or xanthine. The assay mixture contained 60 µL of xanthine oxidase solution, 117 µL of potassium phosphate buffer, 3 µL of test sample, and 100 µL of substrate in a 96-well microplate. Immediately after mixing, changes in absorbance at 290 nm were recorded for 3 min at 37 °C. Michaelis–Menten constant (*K_m_*) and maximum velocity (*V_max_*) were calculated from the linear equation of the Lineweaver–Burk plot. The inhibition pattern was then classified as uncompetitive, noncompetitive, competitive, or mixed-type inhibition [38,49,63,64].

### 3.7. Molecular Docking

Three-dimensional ligand files for maltol and the positive controls, febuxostat and allopurinol, were downloaded as SDF format from PubChem. The xanthine oxidase crystal complex co-crystallized with febuxostat (PDB ID: 1N5X; resolution: 2.80 Å) was selected from the Protein Data Bank for docking analysis. The co-crystallized ligand was removed from the protein structure prior to docking. Polar hydrogens were then added using AutoDockTools (version 1.5.7). The prepared protein was then converted to PDBQT format. Because the molybdenum cofactor could not be properly recognized and parameterized during receptor preparation in AutoDockTools, it was excluded from the docking model. The grid box was centered at coordinates (96, 52, 38) with dimensions of 40 × 40 × 40 Å [53]. Docking simulations were conducted using AutoDock Vina (version 1.2.x) [65], with 20 independent runs for each ligand. The binding affinities were expressed as mean ± standard deviation (SD) based on the results from the 20 runs. The RMSD between the redocked and crystallographic poses was calculated in PyMOL (version 3.1.6.1) by pairwise fitting of 22 heavy atoms using the pair_fit command. Docking poses and interactions were visualized using Discovery Studio Visualizer (DSV).

### 3.8. SwissADME and ProTox 3.0 Analysis

SwissADME and ProTox 3.0 are useful web-based platforms for predicting the physicochemical properties, pharmacokinetics, and toxicity profiles of drug candidates. Both tools analyze the uploaded chemical structures of maltol and allopurinol to evaluate drug-likeness and ADME-related properties, while ProTox 3.0 provides more detailed toxicity endpoints. Allopurinol exists in multiple tautomeric forms, and the keto tautomer was selected in this study based on its representation in the prediction database.

### 3.9. Statistical Analysis

Experimental data were analyzed using SPSS (Chicago, IL, USA, Version 16). Group differences were further examined using Scheffe’s post hoc multiple comparison procedure at a 95% confidence level.

## 4. Conclusions

Gout is a metabolic disorder associated with disrupted purine metabolism, resulting in hyperuricemia and monosodium urate crystal deposition in the joints. A daily diet with purine-rich foods (shellfish, red meat, organ meats), beer and sugary drinks can easily lead to gout, and the development of anti-gout agents with fewer adverse effects is urgently needed. The effects of *A. kawakamii* leaf extract, ethyl acetate fraction, and its constituent on xanthine oxidase inhibition were investigated in this study. The EAF was the most effective at inhibiting xanthine oxidase activity among all tested fractions, showing IC_50_ values of 48.76 μg/mL for hypoxanthine and 66.70 μg/mL for xanthine as substrates. Maltol, obtained and characterized from the ethyl acetate fraction, exhibited the inhibitory of xanthine oxidase for both substrates. The IC_50_ values of maltol against xanthine oxidase were 26.67 μg/mL and 33.18 μg/mL when using hypoxanthine and xanthine as substrates, respectively. Enzyme kinetic analysis demonstrated that maltol exhibited mixed-type inhibition against xanthine oxidase using both substrates. In silico docking predicted that maltol can bind to xanthine oxidase with a binding affinity of −6.5 kcal/mol, which is comparable to that of allopurinol, while showing no predicted hepatotoxicity. The results revealed that *A. kawakamii* leaf extract, ethyl acetate fraction, and maltol have potential as potential natural agents targeting xanthine oxidase for gout treatment, although further research is required to confirm their clinical feasibility.

## Figures and Tables

**Table 1:** Inhibition rates of *A. kawakamii* extract and four fractions against xanthine oxidase.

Specimen	Inhibition Rates (%)	
	Hypoxanthine as the Substrate	Xanthine as the Substrate
ALE	44.40 ± 2.08	31.07 ± 3.60
HXF	– *	–
EAF	100.00 ± 0.00	80.82 ± 1.98
BUF	19.36 ± 0.97	30.40 ± 8.99
HOF	45.20 ± 5.56	70.13 ± 7.81
Allopurinol **	100.00 ± 0.00	100.00 ± 0.00

ALE: *A. kawakamii* leaf extract, HXF: *n*-hexane fraction, EAF: ethyl acetate fraction, BUF: *n*-butanol fraction (BUF), and HOF: aqueous fraction; concentration: 100 μg/mL; results are mean ± SD (*n* = 3). – *: no effect; ** Positive control.

**Table 2:** IC_50_ values of leaf extract, ethyl acetate fraction (EAF), and maltol against xanthine oxidase.

Specimen	IC_50_ (μg/mL)	
	Hypoxanthine as the Substrate	Xanthine as the Substrate
Leaf extract	136.57 ± 1.79 ^a^	152.04 ± 6.92 ^A^
EAF	48.76 ± 1.68 ^b^	66.70 ± 1.64 ^B^
Maltol	26.67 ± 0.43 ^c^	33.18 ± 1.12 ^C^
Allopurinol *	0.23 ± 0.04 ^d^	0.30 ± 0.01 ^D^

Data are presented as mean ± SD (*n* = 3). * Positive control. Values sharing different superscript letters (a–d; A–D) within the same substrate group differ significantly at *p* < 0.05 based on Scheffe’s test.

**Table 3:** ^1^H and ^13^C NMR data of maltol.

Position	^13^C	^1^H
2	148.8	
3	143.1	
4	172.9	
5	112.9	6.39 (1H, d, *J* = 5.55 Hz)
6	154.2	7.69 (1H, d, *J* = 5.55 Hz)
C2-CH_3_	14.3	2.34 (3H, s)

**Table 4:** Kinetic parameters of allopurinol-mediated xanthine oxidase inhibition.

Substrate	Kinetic Parameter	Concentration (μg/mL)						Potential	Inhibition Type
		0.000	0.156	0.313	0.625	1.250	2.500		
Hypoxanthine	V_max_	0.00025	0.00025	0.00025	0.00024	0.00024	0.00023	― *	Competitive
	K_m_	0.00178	0.01003	0.01655	0.02007	0.02576	0.03277	↑ **	
Xanthine	V_max_	0.00027	0.00027	0.00026	0.00026	0.00025	0.00026	―	Competitive
	K_m_	0.00165	0.00467	0.01369	0.02060	0.02731	0.03867	↑	

*: constant; **: increasing.

**Table 5:** Kinetic parameters of ethyl acetate fraction-mediated xanthine oxidase inhibition.

Substrate	Kinetic Parameter	Concentration (μg/mL)						Potential	Inhibition Type
		0	20	40	50	60	80		
Hypoxanthine	V_max_	0.00030	0.00027	0.00025	0.00024	0.00021	0.00017	↓ *	Mixed type
	K_m_	0.00047	0.00376	0.00705	0.00796	0.01239	0.01738	↑ **	
Xanthine	V_max_	0.00027	0.00027	0.00026	0.00026	0.00025	0.00026	― ***	Competitive
	K_m_	0.00165	0.00467	0.01369	0.02060	0.02731	0.03867	↑	

*: decreasing; **: increasing; ***: constant.

**Table 6:** Kinetic parameters of maltol-mediated xanthine oxidase inhibition.

Substrate	Kinetic Parameter	Concentration (μg/mL)						Potential	Inhibition Type
		0	10	20	40	60	80		
Hypoxanthine	V_max_	0.00030	0.00027	0.00025	0.00024	0.00021	0.00017	↓ *	Mixed
	K_m_	0.00047	0.00376	0.00705	0.00796	0.01239	0.01738	↑ **	
Xanthine	V_max_	0.00036	0.00032	0.00027	0.00021	0.00017	0.00014	↓	Mixed
	K_m_	0.00345	0.00507	0.00638	0.00825	0.00954	0.01025	↑	

*: decreasing; **: increasing.

## Data Availability

The data are available from the corresponding author on reasonable request.

## Supplementary Materials

The following supporting information can be downloaded at: https://www.mdpi.com/article/10.3390/ph19071100/s1, Figure S1. Two-dimensional interaction diagrams of the top-ranked docking poses of (a) febuxostat, (b) allopurinol, and (c, d) maltol with xanthine oxidase. Table S1. Binding affinity of febuxostat, allopurinol, and maltol to xanthine oxidase. Table S2. Prediction of the molecular descriptors of allopurinol and maltol. Table S3. Prediction of pharmacokinetic profile of allopurinol and maltol. Table S4. Prediction of toxicity of allopurinol and maltol.
