# Supplementary material for: In Silico Molecular Docking Studies and Xanthine Oxidase Inhibitory Activity of Abies kawakamii Leaf Extract and Its Constituent
Source: Pharmaceuticals (Basel). 2026 Jul 17;19(7):1100. doi: 10.3390/ph19071100 (PMC13414989; doi:10.3390/ph19071100)
Supplement: Supplementary file 1 [file pharmaceuticals-19-01100-s001.zip › pharmaceuticals-4387957-supplementary.pdf]

Supporting Information

In Silico Molecular Docking Studies and Xanthine Oxidase Inhibitory Activity of *Abies kawakamii* Leaf Extract and Its Constituent

Chi-Ya Huang [1], Pei-Ling Yen [2], Li-Sheng Hsu [3], Jinn-Guan Low [3], Yu-Mei Huang [4], Shou-Ling Huang [5], Chun-Han Ko [3] and Hui-Ting Chang [3,*]

[1] Agricultural Technology Research Institute, Hsinchu 300110, Taiwan; r99625047@ntu.edu.tw
[2] Program in Specialty Crops and Metabolomics, Academy of Circular Economy, National Chung Hsing University, Nantou 54071, Taiwan; plyen@dragon.nchu.edu.tw
[3] School of Forestry and Resource Conservation, National Taiwan University, Taipei 10617, Taiwan; r97625041@ntu.edu.tw (L.-S.H.); r13625045@ntu.edu.tw (J.-G.L.); chunhank@ntu.edu.tw (C.-H.K.)
[4] Institute of Environmental and Occupational Health Sciences, National Taiwan University, Taipei 10055, Taiwan; d95841007@ntu.edu.tw
[5] Instrumentation Center, College of Science, National Taiwan University, Taipei 10617, Taiwan; shoul-ing@g.ntu.edu.tw
* Correspondence: chtchang@ntu.edu.tw; Tel.: +886-2-3366-5880

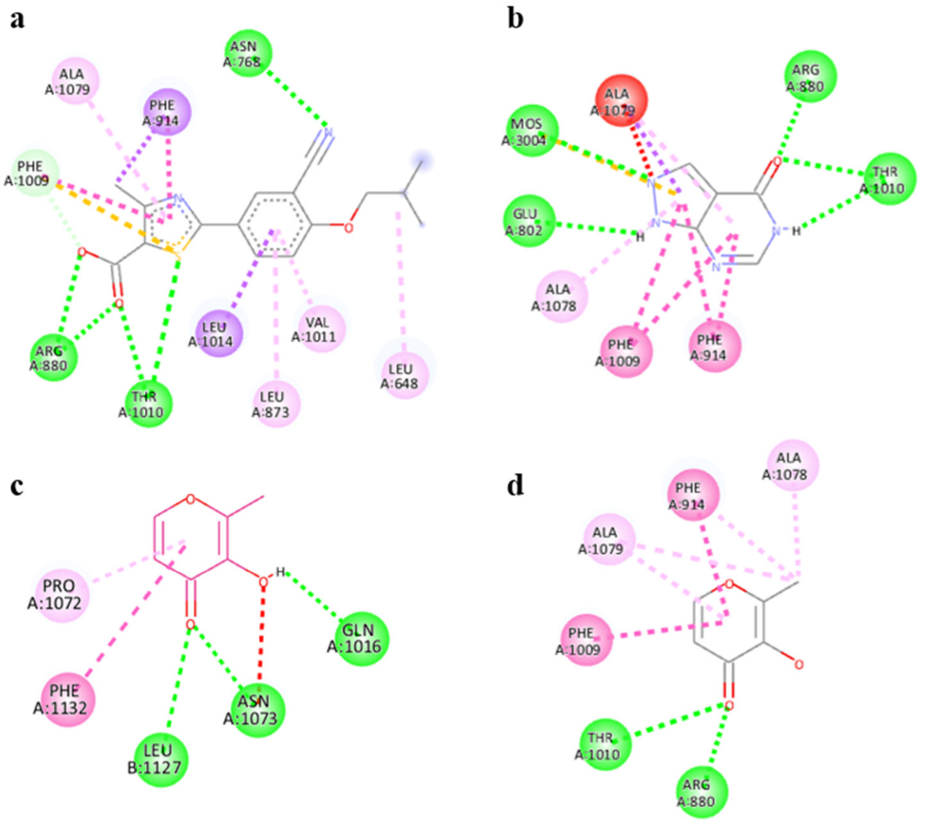

Figure S1. Two-dimensional interaction diagrams of the top-ranked docking poses of (a) febuxostat, (b) allopurinol, and (c, d) maltol with xanthine oxidase. Green dashed lines represent hydrogen bonds; pink lines represent π–π and π–alkyl interactions; purple lines represent π–sigma interactions; gold lines represent π–sulfur interactions; and red lines represent unfavorable donor–donor interactions.

Table S1. Binding affinity of febuxostat, allopurinol, and maltol to xanthine oxidase.

Chemical	Binding affinity (kcal/mol)
Febuxostat	−8.5 ± 0.3
Allopurinol	−7.0 ± 0.1
Maltol	−6.5 ± 0.1

Table S2. Prediction of the molecular descriptors of allopurinol and maltol.

Chemical	MW	NRB	HBA	HBD	MR	TPSA	Log S	Log P
Allopurinol	136.11	0	3	2	34.51	74.43	-0.93	0.01
Maltol	126.11	0	3	1	31.97	50.44	-1.17	0.55

MW: molecular weight; NRB: number of rotatable bonds; HBA: hydrogen bond acceptors; HBD: hydrogen bond donors; MR: molar refractivity; TPSA: topological polar surface area (Å); Log S: logarithm of solubility (ESOL); Log P: average of the logarithm of the partition coefficient (Consensus).

Table S3. Prediction of pharmacokinetic profile of allopurinol and maltol.

Chemical	CYP isoforms inhibition	GI ab	BBB	P-gp	Log *Kp*
Allopurinol	No	High	No	No	-7.61
Maltol	No	High	Yes	No	-7.01

CYP isoforms inhibition: inhibition of the main CYP450 isoforms (1A2, 2C9, 2C19, 2D6 and 3A4); GI ab: gastrointestinal absorption; BBB p: blood-brain barrier permeability; P-gp: inhibitor or substrate for P-glycoprotein; Log *Kp*: skin permeation (cm/s).

Table S4. Prediction of toxicity of allopurinol and maltol.

Chemical	Hepato	Neuro	Nephro	Respi	Cardio	Carcino	Immuno	Mutagen	Cyto
Allopurinol	+ (0.73)	+ (0.69)	+ (0.69)	+ (0.60)	- (0.93)	- (0.52)	- (0.97)	- (0.81)	- (0.89)
Maltol	- (0.60)	- (0.75)	+ (0.53)	- (0.58)	- (0.68)	+ (0.51)	- (0.95)	+ (0.69)	- (0.83)

Prediction with (probability, %): + active; - inactive. Hepato: Hepatotoxicity; Neuro: Neurotoxicity; Nephro: Nephrotoxicity; Respi: Respiratory toxicity; Cardio: Cardiotoxicity; Carcino: Carcinogenicity; Immuno: Immunotoxicity; mutagen: Mutagenicity; Cyto: Cytotoxicity.
